# Capsaicinoid Profiles, Phenolic Content, and Antioxidant Properties of Chili Peppers Grown in Urban Settings

**DOI:** 10.3390/ijms26104916

**Published:** 2025-05-20

**Authors:** Malak Alghamdi, Thirumurugan Rathinasabapathy, Slavko Komarnytsky

**Affiliations:** 1 Plants for Human Health Institute, NC State University, 600 Laureate Way, Kannapolis, NC 28081, USA; malgham@ncsu.edu (M.A.); trathin@ncsu.edu (T.R.); 2 Department of Food, Bioprocessing, and Nutrition Sciences, NC State University, 400 Dan Allen Drive, Raleigh, NC 27695, USA

**Keywords:** *Capsicum cultivars*, capsaicin, phenolic compounds, antioxidant activity, urban gardening, morphological traits, phytochemical variability

## Abstract

The *Capsicum* genus, native to the Americas and cultivated worldwide for culinary and medicinal purposes, includes five domesticated species with diverse fruit characteristics, pungency, and phytochemical profiles. However, the influence of casual urban backyard growing conditions on these traits remains unknown. In this study, we first assessed morphological production traits of 11 popular pepper cultivars over two growing seasons to establish a consistent baseline for cultivar performance. Next, we evaluated capsaicinoid and phenolic profiles of 47 pepper cultivars, which contribute to their pungency and antioxidant properties. Capsaicinoid profiles revealed species-specific ratios of capsaicin, dihydrocapsaicin, and nordihydrocapsaicin, with *C. annuum* and *C. baccatum* displaying an average 64:30:6 profile, *C. chinense* and *C. frutescens* showing a capsaicin-dominant 73:25:2 profile, and *C. pubescens* expressing a distinct dihydrocapsaicin-dominant 34:60:6 profile. Antioxidant activity positively correlated with capsaicinoid content (ABTS: R^2^ = 0.8264, *p* < 0.0001; FRAP: R^2^ = 0.8117, *p* < 0.0001), with *C. chinense* (Carolina Reaper) exhibiting the highest activity (FRAP = 111.8 µM TE/g). In LPS-activated macrophages, all cultivars suppressed nitric oxide production both at the enzymatic (66–89%, *p* < 0.001) and gene expression levels (4.2 to 5.3-fold reduction, *p* < 0.05). Interleukin IL-1β expression was upregulated (3.8 to 12.9-fold, *p* < 0.001), while no significant effects were noted on Cox-2, IL-6, and MCP-1 mRNA levels. These results provide novel insights into the molecular and biochemical adaptations of peppers grown in urban environments and underscore the importance of optimizing cultivation conditions to maximize their bioactive potential and health benefits.

## 1. Introduction

The *Capsicum* genus, part of the *Solanaceae* family, comprises a diverse group of pepper species native to the Americas [1] and widely cultivated worldwide for their culinary and medicinal uses [2]. The Andean region of western to northwestern South America is considered the ancestral range of the genus [3]. However, three major domesticated species, *Capsicum annuum* L. (chili), *Capsicum frutescens* L. (tabasco), and *Capsicum chinense* Jacq. (habanero), were likely brought into cultivation in central-eastern Mexico [4] as early as 6000 BCE [5], with their abundance increasing notably between 600 and 1200 AD [6]. The remaining domesticated species, *Capsicum baccatum* L. (aji) and *Capsicum pubescens* Ruiz & Pav. (rocoto), were likely introduced from South America, where they were domesticated in the mid- and high-altitude Andes, respectively [7]. The triple origin of domesticated *Capsicum* species has been supported by several molecular phylogenetic studies, including high-resolution chloroplast genome analyses [8] and genome-wide RAD-seq data [9].

Although many wild and some domesticated pepper forms grow as woody perennials that tolerate marginal chilling or freezing temperatures, modern cultivars are predominantly grown seasonally as herbaceous annuals [10]. The genetically narrow range of modern F1 lines, bred for enhanced disease resistance and shelf life, is cultivated at scale by modern agricultural systems [11]. However, hundreds of heirloom cultivars and endemic landraces, exhibiting remarkable diversity in fruit characteristics (shape, color), pungency (measured in Scoville Heat Units or SHU), and aromas (sweet, grassy, smoky, fruity, floral, or intensely spicy), remain highly valued by small farmers, traditional gardeners, and pepper enthusiasts worldwide [12]. Pepper cultivars accumulate a wide range of phytochemicals with the potential to improve human health, including vitamin C [13], carotenoids [14], and phenolic compounds [15]. Phenolics significantly enhance the nutritional and functional value of peppers, with total phenolic content, ranging from 3 to 7 mg gallic acid equivalents per gram of dry weight (GAE/g DW) [16]. The phenolic profile of peppers is primarily composed of flavonoids (quercetin, luteolin, and apigenin), along with chlorogenic acid, caffeic acid, and ferulic acid, which contribute to immune modulation and cardiovascular protection [17]. These compounds also exhibit strong antioxidant properties, effectively scavenging reactive oxygen species (ROS) that are implicated in the development of chronic diseases [18].

Capsaicinoids are a unique group of phytochemicals found exclusively in peppers, classified as both phenolics, due to the hydroxyl group attached to an aromatic ring in their core structure, and alkaloids, owing to the nitrogen-containing amide moiety [19]. These compounds are primarily produced in the glands of the placental dissepiment and are responsible for the heat and pungency of different pepper cultivars [20]. The high capsaicinoid concentration in the placenta deters mammals and other herbivores from consuming the fruit, while birds, unaffected by capsaicinoids, facilitate seed dispersal [21]. The pungency of pepper cultivars is defined by their capsaicinoid profiles, categorized as super-hot (300,000+ SHU), extra-hot (100,000–300,000 SHU), hot (30,000–100,000 SHU), medium (3000–30,000 SHU), mild (500–3000 SHU), and non-pungent (0–500 SHU), with pure capsaicin rated at 16,100,000 SHU [22].

The burning sensation elicited by capsaicinoids is mediated by the TRPV1 receptor [23] and is exploited in culinary applications [24], personal safety [25], and pain management [26]. Capsaicin and dihydrocapsaicin are the most potent capsaicinoids, with modifications to the fatty acyl chain decreasing potency while altering the perceived duration and intensity of the burn [27]. Topically, capsaicin is widely used for its analgesic properties in managing chronic pain conditions, such as lower back pain, by desensitizing sensory neurons and reducing the transmission of pain signals [28]. Its activation of the TRPV1 receptor also contributes to reducing adipogenesis and promoting energy expenditure, thereby supporting the prevention of obesity and related metabolic disorders [29]. Additionally, TRPV1-mediated pathways enhance endothelial nitric oxide production, leading to improved vasorelaxation and reduced vascular resistance [30].

Capsaicin accumulation in pepper cultivars is influenced by genetic factors, including the activity of the phenylpropanoid and branched-chain fatty acid pathways, as well as environmental conditions [31]. Higher temperatures, increased sunlight, moderate water stress, and advanced ripening stages enhance capsaicin content by activating secondary metabolism [32]. Soil properties, such as pH, nutrient availability, and organic matter, further affect capsaicinoid profiles [33]. Despite the growing global interest in cultivating hotter peppers [34], data on pungency levels from small garden and urban environments remain scarce. While extensive research has focused on optimizing pepper cultivation in commercial agricultural systems, little is known about how peppers perform in urban and small-scale garden environments. These settings often present unique challenges [35], including limited space, suboptimal soil quality, microclimate variations, and inconsistent resource availability, which can affect yield, quality, and phytochemical composition [36]. Moreover, the growing interest in local, self-sustained food production and urban gardening initiatives highlights the need to understand how non-commercial cultivation impacts the health-promoting properties of urban crops [37]. For example, raising microgreens in urban settings enhances the phytochemical profiles and antioxidant capacities of edible seeds and improves access to nutrient-dense, bioactive-rich foods in urban environments [38].

The aim of this study was, therefore, to investigate changes in production characteristics of 11 pepper cultivars from five different *Capsicum* species over 2 years of cultivation in small garden space and urban environments, where growing conditions differ from commercial agricultural systems. Furthermore, capsaicinoid profiles and total phenolic content were evaluated in an expanded set of 47 pepper cultivars and correlated with their antioxidant and immunomodulatory properties in macrophages. This allowed us to enhance the understanding of how hobbyist growers and small-scale producers experience the challenges of cultivating hot peppers in non-optimized environments, with a focus on quantifying their bioactive potential and health benefits under these conditions.

## 2. Results

### 2.1. Pepper Fruit Quality Characteristics over Two Growing Seasons

A selected set of 11 popular pepper cultivars from five different *Capsicum* species was grown in typical urban settings (suburban backyard) over the course of two years to determine the variation in the pepper fruit set and quality characteristics associated with casual growing conditions and random weather patterns. Fruit size, weight, and moisture content showed little variability over the period of the study, suggesting that all cultivars were well established (Table 1).

### 2.2. Capsaicinoid Profiles of 47 Pepper Cultivars Grown During Year 2

The expanded set of 47 chili pepper cultivars from five different *Capsicum* species was grown during year 2 of the study to evaluate their capsaicinoid profiles and pungency (Table 2) and correlate them to the total phenolic content and biological activity associated with the antioxidant and immunomodulatory properties.

The cultivars from the *C. annuum* genetic background included popular Sweet Banana (28 SHU) and Vezena Piperka (113 SHU), as well as medium-hot Shishito and Jalapeno peppers (18,000–19,000 SHU). The Scoville values for this group peaked at 123,321 SHU, with capsaicin dominating the profile at a ratio of 68:27:5 (C:DHC:NDHC).

The cultivars from *C. baccatum* complex included peppers selected to contain no measurable pungency, such as Aji Delight, Aji Cambuci or Mad Hatter, with the latter two often referred to as the same or very closely related cultivars (0–370 SHU). The majority of the *baccatum* peppers fell into the medium category (14,000–36,000 SHU). The popular Lemon Drop variety showed the highest Scoville value of the group at 57,000 SHU. The average capsaicinoid profile closely resembled that of the *annuum* complex, with a ratio of 60:32:8.

The *C. chinense* cultivars were characterized by predominantly hot to super-hot pungency scores that reflected their popularity as extra-pungent chili peppers. Most of the scores were observed in the 70,000–370,000 SHU range, with Dragon Breath (458,029 SHU) and Carolina Reaper (518,299 SHU) at the higher end of the spectrum. The CGN 21500 (Sunset Peach) accession that is closely related to the wild *C. chinense* plants showed 160,000–200,000 SHU Scoville scores typical of the *chinense* complex. Habanero Sweet (64 SHU) and Tobago Seasoning (129 SHU) were the non-pungent *chinense* cultivars. The Biquinho group that is very popular as a pickling pepper of red, yellow, and white coloration, showed a moderate pungency value (34,500–54,700 SHU), as well as a successful attempt to breed a sweet cultivar (97 SHU). The average capsaicinoid profile for the *chinense* group was skewed toward higher capsaicin accumulation and lower nordihydrocapsaicin levels, with a ratio of 71:26:2.

A single cultivar from both the *C. frutescens* complex (Wiri Wiri at 167,000 SHU) and the *C. pubescens* complex (Rocoto Red at 53,000 SHU) was found within the Scoville ranges appropriate for these species. The capsaicinoid profile of *C. frutescens* resembled that of the *chinense* complex, with a ratio of 74:25:1, while *C. pubescens* showed a distinct tendency to accumulate dihydrocapsaicin, with a ratio of 34:60:7. Several cultivars from different complexes were tested as two independent accessions to evaluate the within-year variability, which was rather high at 21–35%, depending on the cultivar (Table 2).

### 2.3. Total Phenolic Content

The phenolic content of 47 chili peppers from the total methanolic extracts varied significantly across species and cultivars (Table 3). Among the *C. annuum* species, cultivars such as Jalapeno (1.7–1.9 mg/g) exhibited higher phenolic content. In *C. baccatum*, cultivars such as Sugar Rush Peach (1.5 mg/g) had moderate phenolic levels, while others, such as Aji Delight and Oro de Ecuador (0.7–0.8 mg/g) had relatively low values. *C. chinense* cultivars, notably Carolina Reaper (3.1 mg/g) and Dragon Breath (2.4 mg/g), showed the highest total phenolic content among the cultivars studied. On the other hand, *C. frutescens* and *C. pubescens* cultivars demonstrated moderate phenolic levels. Overall, the phenolic content in the analyzed chili peppers displayed a broad spectrum, correlating with the diversity in capsaicinoid profiles.

### 2.4. Antioxidant Activity

The antioxidant capacities of 47 chili peppers from the total methanolic extracts were assessed using ABTS, DPPH, and FRAP assays, revealing notable differences across cultivars and species (Table 3). In *C. annuum*, the Cayenne cultivar exhibited relatively high antioxidant activity, particularly in the ABTS (16.2 µM TE/g) and FRAP (18.4 µM TE/g) assays, reflecting its robust phenolic content. The Ethiopian Brown cultivar had moderate antioxidant values, aligning with its capsaicinoid concentration. Conversely, Banana Sweet, with negligible capsaicinoid levels, displayed the lowest antioxidant activity.

Among *C. baccatum* peppers, the Lemon Drop cultivar demonstrated the highest antioxidant potential, consistent across ABTS (7.3 µM TE/g) and FRAP (7.2 µM TE/g) assays. Aji Mango and Sugar Rush Peach also exhibited notable activity, with phenolic content positively correlating to their antioxidant capacity. Lower values were observed in Aji Delight and Aji Cambuci, likely due to reduced phenolic and capsaicinoid levels. In *C. chinense*, the Carolina Reaper stood out with the highest antioxidant activity across all assays, particularly FRAP (111.8 µM TE/g), correlating with its high capsaicinoid levels. Cultivars such as Dragon Breath and Hallows Eve also displayed strong activity.

For *C. frutescens*, Wiri Wiri demonstrated substantial antioxidant capacity, especially in FRAP (20.8 µM TE/g), correlating with its high capsaicinoid content. Lastly, in *C. pubescens*, Rocoto Red exhibited moderate activity, consistent with its phenolic concentration. Overall, the capsaicinoid content of all pepper cultivars positively correlated with both ABTS (R^2^ = 0.8264, *p* < 0.0001) and FRAP (R^2^ = 0.8117, *p* < 0.0001) antioxidant scores, emphasizing the potential health benefits of pungent varieties. The correlation between the total phenolic content and the antioxidant capacity of individual peppers was only moderate (R^2^ = 0.5726–0.6137, *p* < 0.0001).

### 2.5. Nitric Oxide Production in Macrophages

**Figure 1:**
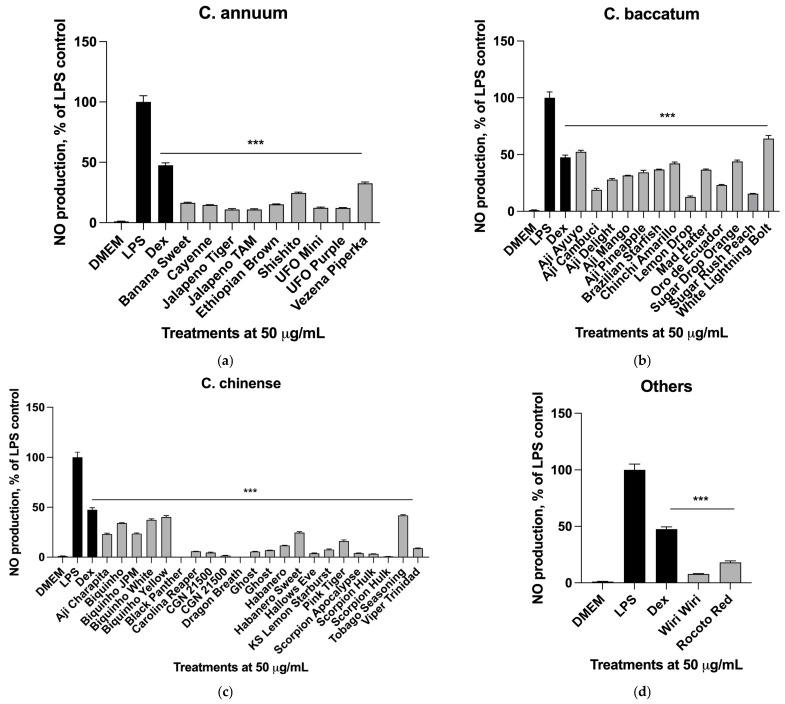
Effects of total extracts of pepper cultivars including (**a**) *C. annuum*, (**b**) *C. baccatum*, (**c**) *C. chinense*, and (**d**) *C. frutescens* and *C. pubescens* on nitric oxide production in activated macrophages. Cells were treated with target samples at 50 µg/mL and the inflammatory response was induced with 1 µg/mL LPS for 6 h (*n* = 3). The assay was validated with 10 µM dexamethasone as a reference control. Changes in nitrite concentration as an indirect measure of nitric oxide production were reported as mean ± SEM relative to the LPS controls (*** *p* < 0.001).

The analysis of nitric oxide release in the LPS-activated RAW 264.7 macrophages treated with 47 total chili pepper methanolic extracts revealed some variability in the inflammatory response that clustered among the different species of the *Capsicum* genus. There was little to no variation within sweet or pungent cultivars of *C. annuum*, all tested varieties suppressed nitric oxide release from the activated macrophages in the range of 66–89% and average of 83.1 ± 5.5% (Figure 1a).

The *C. baccatum* cultivars showed weaker responses in the same assay. The inhibition of nitric oxide release ranged between 36 and 87% and averaged 66.1 ± 14.7% (Figure 1b). The white fruited cultivar White Lightning Bolt showed the weakest suppression of nitric oxide release, suggesting that pigments responsible for the pepper color significantly contribute to the overall NO-suppressing activity of the fruit.

The *C. chinense* varieties showed the highest NO-reducing properties. The inhibition of nitric oxide release ranged between 58 and 100% and averaged 86.1 ± 13.9% (Figure 1c). The suppression was highest among the extra-hot varieties, with Black Panther (100%), Dragon Breath (99%), Scorpion Hulk (99%), Scorpion Apocalypse (96%), and Carolina Reaper (95%) showing the most potent immune-modulating properties. The two cultivars in *C. frutescens* and *C. pubescens* species showed comparable effects at 87.1 ± 7.3% inhibition (Figure 1d).

### 2.6. Gene Expression Profiles in Activated Macrophages

**Figure 2:**
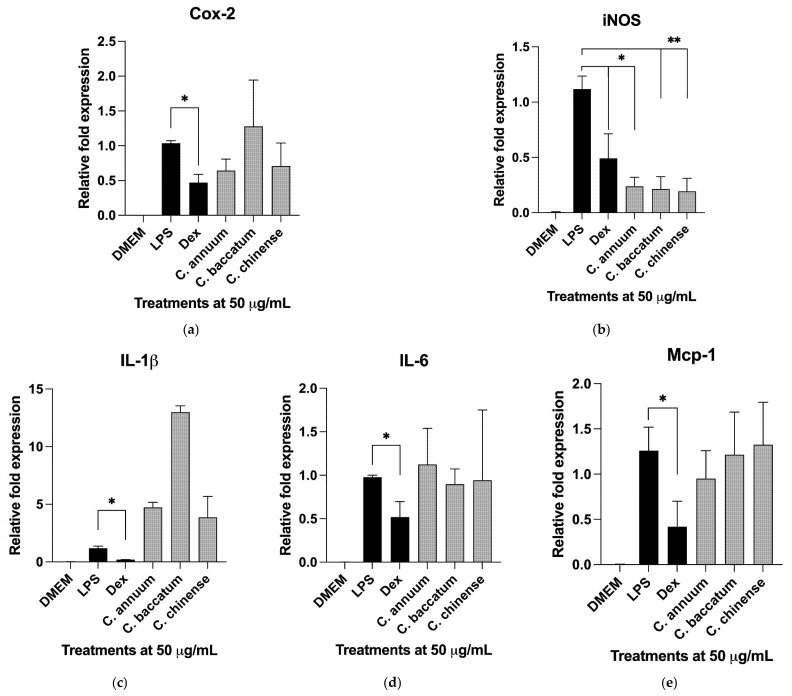
Inflammatory gene expression profiles in the LPS-activated macrophages focusing on (**a**) cyclooxygenase-2 (COX-2), (**b**) inducible nitric oxide synthase (iNOS), (**c**) interleukin 1β (IL-1β), (**d**) interleukin 6 (IL-6), and (**e**) monocyte chemoattractant protein-1 (MCP-1) in duplicate (*n* = 2). The assay was validated with 10 µM dexamethasone as a reference control. Data were reported as mean ± SEM and analyzed by ANOVA followed by Dunnett multiple comparisons test. Fold changes in gene expression are reported as means relative to the LPS-induced control (* *p* < 0.05, ** *p* < 0.01).

LPS activation resulted in a marked increase in the short-lived core cluster of effector cytokines and chemokines that drive the initial pro-inflammatory responses following the LPS challenge. This network centers on NF-κB-mediated activation of the IL-1β/IL-6 and IL-18/MCP-1 signaling to upregulate cyclooxygenase-2 (COX-2) and inducible nitric oxide synthase (iNOS) in macrophages [39]. To check the activity of these pathways, *C. annuum*, *C. baccatum*, and *C. chinense* cultivars were tested for their ability to modulate imflammatory gene expression profiles in the LPS-activated macrophages. *C. frutescens* and *C. pubescens* were excluded from the analysis because they were represented by only one cultivar. In line with nitric oxide release (Figure 1), pepper varieties showed suppression of inducible nitric oxide synthase (iNOS) expression for all species (*p* < 0.05 and *p* < 0.01) (Figure 2b). However, the gene expression biomarkers for Cox-2, IL-6 and MCP-1 signaling were not affected, and IL-1β expression was induced (3.8 to 12.9-fold increase, *p* < 0.001), suggesting the presence of alternative regulatory mechanisms.

## 3. Discussion

Growing peppers is a rewarding, low-cost activity that promotes stress relief, fosters a connection with nature, and provides a sense of accomplishment [34]. Both commercial producers and home gardeners find fulfillment in blending ancient chili cultivation practices with modern agricultural advances, making the process both meaningful and exciting [40]. Urban gardeners are often drawn to culturally significant pepper varieties, such as habaneros or bird’s eye chilies, fostering a connection to their heritage or global culinary traditions [41]. Additionally, some urban growers monetize their hobby by selling fresh peppers, homemade hot sauces, or seeds at local markets or through online platforms, turning their passion into a small business [42].

Urban gardeners frequently push boundaries in small spaces, experimenting with growing conditions and techniques to optimize pepper production in their settings. While the literature is rich with data from controlled academic or commercial environments [43], this study offers a distinct perspective on pepper cultivation in urban backyards, where environmental conditions are more variable. The morphological traits of the pepper cultivars used in this study, including fruit length, width, fresh weight, and moisture content, exhibited minimal variability across two growing years (Table 1). This consistency suggested that most cultivars were well adapted to urban conditions. Furthermore, the limited variation highlights the genetic stability of these cultivars, which may be beneficial for growers aiming to meet specific consumer preferences.

The capsaicinoid profiles revealed capsaicin as the primary contributor to pungency, with lesser contributions from dihydrocapsaicin and nordihydrocapsaicin (Table 2). The consistent C:DHC:NDHC ratios across most species suggest that environmental factors primarily influenced the total capsaicinoid yield rather than the relative composition. Cultivars such as Carolina Reaper and Dragon Breath, which exhibited high SHU values, showed potential for pharmaceutical and pest control applications [44]. However, the uncontrolled urban growing conditions may limit their capsaicinoid production, as the highest pungency levels observed (450,000–520,000 SHU) were notably lower than the 1.5+ million SHU reported in highly controlled, optimized environments [45]. Variation in capsaicinoid levels may also be influenced by environmental factors, such as temperature fluctuations, soil composition, and sunlight exposure, all of which affect capsaicinoid biosynthesis [46], as supported by similar findings in other studies [47]. However, since this study did not assess soil parameters, the role of nutrient availability and soil chemistry in pungency variation remains unclear, highlighting the need for future research that includes detailed soil analysis across multiple growing seasons [48].

Capsaicinoids, due to their dual alkaloid and phenolic nature, significantly contributed to the antioxidant properties of the analyzed peppers. Capsaicinoid levels strongly correlated with ABTS (R^2^ = 0.8264, *p* < 0.0001) and FRAP (R^2^ = 0.8117, *p* < 0.0001) antioxidant scores, but showed no significant association with the DPPH score (R^2^ = 0.2839). Cultivars with higher capsaicinoid concentrations, such as Carolina Reaper and Dragon Breath, also exhibited the highest antioxidant activity (FRAP 98–112 μM TE/g, ABTS 65–78 μM TE/g dry weight). These findings are consistent with previous studies linking capsaicinoids to oxidative stress reduction [49]. Additionally, the data revealed a moderate positive correlation between pungency and total phenolic content (R^2^ = 0.4579), suggesting that phenolics may also contribute to the observed biological activity. Among the phenolic compounds identified in peppers, luteolin, capsaicin, and quercetin [50], as well as apigenin [51], have previously been shown to enhance their antioxidant properties.

The relationship between capsaicinoids and the inhibition of NO production in LPS-activated macrophages was moderate (R^2^ = 0.3796), suggesting that other phytochemical groups, such as phenolics (R^2^ = 0.3042), and possibly carotenoids (not quantified in this study), also contributed significantly to the NO-reducing effects of the pepper cultivars. Inhibition was observed at both the enzymatic level (Figure 1) and through the suppression of inducible nitric oxide synthase (iNOS) mRNA expression (Figure 2). These findings align with previous reports demonstrating capsaicin-mediated NO inhibition in vascular epithelial cells [52] and macrophages [53]. However, the immunomodulatory effects in this study were not associated with suppression of the core IL-1β/IL-6 and IL-18/MCP-1 signaling networks; instead, IL-1β expression was markedly increased (3.8 to 12.9-fold, *p* < 0.001). While this contrasts with capsaicin’s anti-inflammatory effects on LPS-induced inflammation in human THP-1 cells [54], the findings are consistent with prior observations that capsaicin and IL-1β enhance neurogenic vasodilation [55], capsaicin-treated human epidermal keratinocytes increase IL-1β and IL-1α production [56], and systemic capsaicin pretreatment does not block food-motivated behavior induced by LPS and IL-1β [57]. Recent evidence also suggests that capsaicin-induced heat and mechanical hypersensitivity is mediated by IL-1 signaling [58]. As capsaicinoids bind and activate the transient receptor potential vanilloid 1 (TRPV1) receptor, it is also plausible that the observed effects involve TRPV1-mediated Ca^2+^/CaMKII/Nrf2 signaling, as reported in osteoarthritis models [59]. These potential mechanisms warrant further investigation in future studies.

## 4. Materials and Methods

### 4.1. Plant Materials

Chili peppers from *Capsicum annuum* L., *Capsicum baccatum* L., *Capsicum chinense* Jacq., *Capsicum frutescens* L., and *Capsicum pubescens* Ruiz & Pav. species, together with several non-pungent (sweet) controls, were grown over a two-year period in the 450 m^2^ backyard lot of a typical suburban house (latitude 35.4101° N, longitude 80.5820° W, USDA planting zone 7b/8a, NC, USA). The seeds were purchased from the popular and commonly available chili pepper seed suppliers, including Ferry-Morse, Solana Seeds, Baker Creek, Bohica Pepper Hut, Sherwood’s Seeds, Spitfire, Nikitovka, Pepper Merchant, Etsy, etc.

In February of each year, pepper seeds were soaked on wet paper towels for 3 days before germinating in the seed starting mix (Miracle-Gro, Marysville, OH, USA) using the Guardtree seed nursery bags (Agfabric, Vista, CA, USA) placed on the windowsill with a heat mat set to 26 °C (79 °F) for the first 14 days. Seedlings were grown under the Aerogarden 45W LED grow lights (Boulder, CO, USA) set to the 16 h lights-on and 8 h lights-off cycle. Plants were transplanted to 5-gallon black nursery plastic pots (Hydrofarm, Shoemakersville, PA, USA) and acclimated to the outside growing in April. The April weather was characterized by an average temperature of 14° C (6–20 °C), 114 mm of rainfall, and 58% probability of sunny days. The summer average temperature was 24° C (16–33 °C), 91 mm of rainfall, and 44% probability of sun. The last crop was collected in late October with an average temperature of 12° C (0–28 °C), 84 mm of rainfall, and a 46% probability of sun.

The environmental variables were not controlled as part of the study design to mimic the behavior of an average urban grower. This introduced more variability in the datasets, but it also allowed us to capture real-world growing conditions and their impact on pepper phytochemical profiles. Consequently, the findings were expected to provide a more realistic assessment yield, capsaicinoid content, and phenolic accumulation in heterogeneous urban settings.

A set of 11 popular pepper cultivars was grown during year 1 (2021) and year 2 (2022) to establish a consistent baseline for cultivar performance. Pepper fruits were collected at the time when they reached terminal coloration appropriate for each variety (September–October of each year), photographed, and assessed for their qualitative production characteristics. Production traits were measured from three plants each (*n* = 3–5 fruits). This set of cultivars was chosen because it sufficiently covered all 5 *Capsicum* species and included many cultivars popular in backyard and small garden settings.

An expanded set of 47 pepper cultivars was grown during year 2 (2022) to evaluate capsaicinoid profiles, total phenolics, antioxidants, and immune properties. Mature pepper fruits without green tissues attached were dried in the 40 °C incubator until constant weight, ground into powder, and stored at −80 °C until analysis. This larger subset of cultivars was used to capture high variability in pepper capsaicinoid and phenolic content.

### 4.2. Acetonitrile Extracts and Capsaicin Profiles by HPLC

Dry pepper fruit powders (500 mg) from year 2 were boiled with 5 mL of acetonitrile in a heated water bath at 80 °C for 4 h with agitation. The extracts were centrifuged at 10,000 g for 5 min at room temperature to remove the solids, concentrated to dryness with Rotovapor R210 (Büchi, New Castle, DE, USA), dissolved in 1 mL of acetonitrile, and filtered through a syringe with a 0.45 μm PFTE filter. HPLC-UV analysis was performed using a Shimadzu HPLC system (Kyoto, Japan) equipped with a pump (LC-20AT), an autosampler (SIL-20A), a diode array detector (SPD-M20A), and an automatic column temperature control oven (CTO-20A). Separation was performed on the Restek Ultra C18 column (250 × 4.6 mm, 5 μm) (Bellefonte, PA, USA) at a column temperature of 30 °C, flow rate of 1 mL/min, and injection volume of 20 μL. The binary mobile phase consisted of 10% methanol in water (eluent A) and 100% methanol (eluent B) in a gradient as follows: 0–10 min, 43% eluent A and 57% eluent B; 10–20 min, 32% eluent A and 68% eluent B. UV spectra were monitored at 280 nm. Peaks were identified based on a comparison of retention times and UV spectra with those of authentic standards (Sigma-Aldrich, St. Louis, MO, USA). This procedure was developed based on the previously published method [60].

### 4.3. Metanolic Extracts for Phenolic, Antioxidant, and Cell Culture Assays

Dry pepper fruit powders (500 mg) from year 2 were extracted with 5 mL of 80% methanol in a heated water bath at 60 °C for 4 h. The extracts were centrifuged at 3500 g for 15 min at room temperature, concentrated to remove methanol with a Rotovapor R210 (Büchi, New Castle, DE, USA) and freeze dried with Labconco Freezone 18 (Kansas City, MO, USA).

### 4.4. Total Phenolics Assay

The total phenolic content assay was performed following a modified Folin–Ciocalteu colorimetric method [61]. Dry pepper powders (10 mg) were extracted with 1 mL of acidified 80% methanol in water (0.83% concentrated HCl) for 2 h in the dark, and centrifuged at 10,000× *g* for 10 min. In a 96-well microplate, 140 μL of distilled water, 10 μL of Folin–Ciocalteu reagent (Sigma-Aldrich, St. Louis, MO, USA), and 20 μL of blanks, standards, or samples were added in duplicate (*n* = 2). After 5 min, 30 μL of 20% of sodium carbonate was added and the reactions were kept for an additional 20 min for color to develop. Absorbance at 765 nm against the appropriate blank was determined using the Synergy H1 spectrophotometer (BioTek, Winooski, VT, USA). Gallic acid was used as a standard in a serial dilution of 0.031–2 mg/mL, and phenolic concentrations were reported as gallic acid equivalents (GAE) in mg per 1 g of dry weight.

### 4.5. Free Radical Scavenging (ABTS/TEAC) Assay

The ABTS method of evaluating antioxidant activity of plants relies on scavenging the 2,2′-azino-bis(3-ethylbenzothiazoline-6-sulfonic acid) radical that rapidly reaches a steady state within 30 min in aqueous solutions at a wide pH 2–10 range [62]. In organic solvents, the reaction is slowed down considerably [63]. The ABTS•+ radical was generated in triplicate by mixing 34.4 mg ABTS (7.4 mM) and 6.6 mg potassium persulfate (2.6 mM) in 10 mL of distilled water and stored overnight in the dark. The ABTS•+ radical working solution was prepared by diluting the stock to 49 mL of methanol. Trolox standard was prepared as a series of dilutions of 15.75–1000 μM. In a 96-well microplate, 190 μL of ABTS•+ solution and 10 μL of blanks, standards, or samples were added in triplicate (*n* = 3). The plate was kept in the dark for 30 min, and absorbance at 745 nm against the appropriate blank was recorded using the Synergy H1 spectrophotometer (BioTek, Winooski, VT, USA). The absorbance reduction for the sample was plugged into the Trolox standard curve equation to determine its equivalent Trolox concentration. The results were expressed as μM of Trolox equivalents per 1 g of dry weight (μM TE/1 g DW).

### 4.6. DPPH Antioxidant Assay

The DPPH method measures the reduction of the stable nitrogen radical 2,2-diphenyl-1-picrylhydrazyl [64]. The reaction reaches a steady state in 1–6 h, and underestimates antioxidant activity in the presence of anthocyanins and carotenoids [63]. The solution of DPPH in methanol (0.07 mM) was prepared fresh daily as described [65]. Trolox standard was prepared as a series of dilutions of 15.75–1000 μM. In a 96-well microplate, 270 μL of DPPH• solution and 30 μL of blanks, standards, or samples were added in triplicate (*n* = 3). The plate was kept in the dark for 15 min, and absorbance at 515 nm against the appropriate blank was recorded using the Synergy H1 spectrophotometer (BioTek). The absorbance reduction for the sample was plugged into the Trolox standard curve equation to determine its equivalent Trolox concentration. The results were expressed as μM of Trolox equivalents per 1 g of dry weight (μM TE/1 g DW).

### 4.7. Ferric-Reducing Antioxidant Power (FRAP) Assay

The FRAP assay does not evaluate free radicals, but instead measures reduction of ferric 2,4,6-tripyridyl-s-triazine (TPTZ-Fe^3+^) to a colored product (TPTZ-Fe^2+^) at acidic pH to maintain iron solubility [65]. The FRAP reagent was prepared by mixing 10 volumes of 300 mM acetate buffer (pH 3.6) with 1 volume of 10 mM TPTZ in 40 mM HCl, and with 1 volume of 20 mM ferric chloride as described previously [66]. Trolox standard was prepared as a series of dilutions of 15.75–1000 μM. In a 96-well microplate, 240 μL of FRAP solution and 10 μL of blanks, standards, or samples were added in triplicate (*n* = 3). The plate was kept at 37 °C for 5 min, and absorbance at 620 nm against the appropriate blank was recorded using the Synergy H1 spectrophotometer (BioTek). The absorbance reduction for the sample was plugged into the Trolox standard curve equation to determine its equivalent Trolox concentration. The results were expressed as μM of Trolox equivalents per 1 g of dry weight (μM TE/1 g DW).

### 4.8. Cell Culture

The mouse macrophage cell line RAW 264.7 (ATCC TIB-71) was maintained in DMEM (Life Technologies) supplemented with 10% fetal bovine serum, 100 IU/mL penicillin, and 100 μg/mL streptomycin (Fisher Scientific, Pittsburg, PA, USA) at a density not exceeding 5 × 10^5^ cells/mL. Passages were performed every 3–4 days in 57 cm^2^ cell culture dishes (Nalge Nunc International, Rochester, NY, USA) maintained at 37 °C in a humidified 5% CO_2_ Thermo Forma Series II incubator (Fisher Scientific).

### 4.9. Nitric Oxide Production

For nitric oxide quantification, RAW 264.7 cells were seeded in 96-well plates in triplicate (*n* = 3) at a concentration of 5 × 10^4^ cells/well in a 200 μL culture medium and allowed to adhere for 24 h. The cells were then treated with 50 μg/mL of the target extracts and elicited with 1 μg/mL LPS for an additional 6 h. Nitric oxide released from the stimulated macrophages was quantified using the Greiss reagent system (Promega, Madison, WI, USA) and SynergyH1 microplate reader (BioTek) at 530 nm. The percentage of NO scavenging activity was calculated as [A(LPS control) − A(sample)]/A(LPS control) × 100.

### 4.10. RNA Extraction, cDNA Synthesis, and qPCR

For gene expression studies, RAW 264.7 cells were seeded in 24-well plates at a concentration of 5 × 10^5^ cells/well in a 1 mL culture medium and treated as described. The total RNA was isolated from cells using TRIzol reagent (Life Technologies, Carlsbad, CA, USA) following the manufacturer’s instructions. RNA was quantified using the BioTek SynergyH1/Take 3 plate (Agilent, Santa Clara, CA, USA). The cDNAs were synthesized using 2 μg of RNA for each sample using a high-capacity cDNA Reverse Transcription kit following the manufacturer’s protocol on an ABI GeneAMP 9700 (Life Technologies).

The resulting cDNA was amplified by real-time quantitative PCR using SYBR green PCR master mix (Life Technologies). The amplifications were performed on an ABI 7500 Fast real-time PCR using 1 cycle at 50 °C for 2 min and 1 cycle at 95 °C for 10 min, followed by 40 cycles of 15 s at 95 °C and 1 min at 60 °C. The dissociation curve was completed with 1 cycle of 1 min at 95 °C, 30 s at 55 °C, and 30 s at 95 °C. This study used a following set of primers: β-actin (housekeeping gene), forward primer: 5′-AAC CGT GAA AAG ATG ACC CAG AT-3′, reverse primer: 5′-CAC AGC CTG GAT GGC TAC GT-3′; COX-2, forward primer: 5′-TGG TGC CTG GTC TGA TGA TG-3′, reverse primer: 5′-GTG GTA ACC GCT CAG GTG TTG-3′; iNOS, forward primer: 5′-CCC TCC TGA TCT TGT GTT GGA-3′, reverse primer: 5′-TCA ACC CGA GCT CCT GGA A-3′; IL-1β, forward primer: 5′-CAA CCA ACA AGT GAT ATT CTC CAT G-3′, reverse primer: 5′-GAT CCA CAC TCT CCA GCT GCA-3′; IL-6, forward primer: 5′-TAG TCC TTC CTA CCC CAA TTT CC-3′, reverse primer: 5′-TTG GTC CTT AGC CAC TCC TTC-3′; MCP, forward primer: 5′-CTT CTG GGC CTG CTG TTC A-3′, reverse primer: 5′-GCA GCC TAC TCA TTG GGA TCA-3′. Fold differences in gene expression relative to the LPS-induced controls were analyzed using the ∆∆CT method and normalized with respect to the expression of β-actin.

### 4.11. Statistical Analysis

Data were analyzed by one-way ANOVA followed by Tukey’s multiple-range tests using Prism 10.0 (GraphPad Software, San Diego, CA, USA). All data were presented as means ±  SEM. Significant differences were accepted when the *p*-value was <0.05, and multiple comparisons were used to determine which means were different using the compact letter display based on Tukey’s HSD test.

## 5. Conclusions

Urban gardening fosters a meaningful connection to traditional chili cultivation while promoting wellness and sustainability at the local level, making peppers a compelling subject for both community engagement and scientific research. In this study, we investigated the capsaicinoid and phenolic content of *Capsicum* cultivars grown in backyard urban settings, along with their antioxidant and immunomodulatory effects. Most cultivars exhibited well-defined capsaicinoid profiles and strong antioxidant activity, even under unoptimized conditions. However, achieving peak capsaicinoid production likely requires more controlled and optimized growing environments. Future studies incorporating advanced phenotypic and chemical analyses, soil testing, and diverse urban cultivation methods could expand our understanding of pepper bioactivity and adaptability in small-scale urban agriculture.

## Figures and Tables

**Table 1:** Pepper Fruit Characteristics of Selected Cultivars Grown over 2 Years. Different letters indicate significantly different values based on Tukey’s HSD test (*p* < 0.05).

Species	Cultivar	Internal Code	Growing Year	Length, mm	Width, mm	Fresh Weight, g	Moisture Content, %
***C. annuum***	Banana Sweet	14 (136)	1	95 ± 13 ^a^	32 ± 3 ^a^	27.1 ± 2.8 ^a^	90.7 ± 8.1 ^ab^
			2	106 ± 8 ^a^	35 ± 5 ^a^	33.4 ± 6.1 ^a^	89.1 ± 7.6 ^a^
	Cayenne	93 (138)	1	76 ± 4 ^b^	13 ± 1 ^c^	3.1 ± 0.3 ^e^	80.9 ± 11.4 ^b^
			2	66 ± 2 ^c^	12 ± 1 ^d^	3.6 ± 1.4 ^e^	82.2 ± 9.3 ^c^
	Jalapeno TAM	57	1	66 ± 2 ^c^	25 ± 2 ^b^	15.6 ± 2.3 ^b^	86.6 ± 6.2 ^ab^
			2	66 ± 2 ^c^	24 ± 5 ^b^	14.3 ± 4.1 ^c^	84.2 ± 7.3 b ^c^
***C. baccatum***	Aji Delight	51	1	66 ± 2 ^c^	19 ± 1 ^b^	7.7 ± 0.5 ^d^	78.6 ± 6.3 ^c^
			2	62 ± 12 ^c^	20 ± 2 ^b^	8.2 ± 0.9 ^d^	81.9 ± 4.4 ^c^
	Lemon Drop	52	1	62 ± 5 ^c^	17 ± 1 ^b^	3.5 ± 0.2 ^e^	82.3 ± 12.7 ^b^
			2	59 ± 7 ^c^	16 ± 1 ^c^	3.9 ± 0.4 ^e^	80.5 ± 9.7 ^c^
	Sugar Rush Peach	62	1	67 ± 1 ^c^	22 ± 1 ^b^	9.9 ± 0.6 ^c^	84.9 ± 5.9 ^b^
			2	64 ± 11 ^c^	19 ± 1 ^b^	9.2 ± 1.6 ^d^	82.4 ± 4.0 ^c^
***C. chinense***	Aji Charapita	50	1	13 ± 1 ^f^	10 ± 1 ^c^	0.6 ± 0.02 ^f^	83.5 ± 3.3 ^b^
			2	12 ± 1 ^e^	11 ± 1 ^d^	0.5 ± 0.06 ^g^	81.6 ± 3.9 ^c^
	Biquinho	53	1	24 ± 1 ^f^	13 ± 1 ^c^	1.3 ± 0.1 ^f^	83.8 ± 1.9 ^b^
			2	21 ± 2 ^d^	14 ± 1 ^c^	1.5 ± 0.2 ^f^	85.9 ± 4.5 ^b^
	Habanero	59	1	49 ± 2 ^d^	18 ± 1 ^b^	5.1 ± 0.7 ^e^	87.7 ± 4.4 ^ab^
			2	44 ± 7 ^c^	16 ± 1 ^c^	4.9 ± 0.4 ^e^	88.4 ± 5.1 ^a^
***C. frutescens***	Wiri Wiri	95 (137)	1	16 ± 1 ^f^	15 ± 1 ^bc^	2.4 ± 0.9 ^e^	76.7 ± 13.4 ^c^
			2	14 ± 2 ^d^	17 ± 1 ^bc^	2.2 ± 1.1 ^ef^	81.4 ± 9.8 ^c^
***C. pubescens***	Rocoto Red	49 (154)	1	37 ± 1 ^e^	37 ± 3 ^a^	18.5 ± 1.1 ^b^	93.3 ± 2.7 ^a^
			2	41 ± 4 ^c^	34 ± 6 ^a^	20.9 ± 3.8 ^b^	92.3 ± 4.1 ^a^

**Table 2:** Capsaicinoid Profiles of Chili Peppers Grown During Year 2 of the Study.

Species	Cultivar	Internal Code	C, μg/g ^1^	DHC, μg/g ^2^	NDHC, μg/g ^3^	Pungency, SHU ^4^
***C. annuum***	Banana Sweet	14 (136)	0	0	3	28
	Cayenne	93 (138)	3452	2026	399	91,907
	Jalapeno Tiger	57D	1690	610	148	38,406
	Jalapeno TAM	57E	891	252	35	18,728
	Ethiopian Brown	201	5057	1849	401	114,916
	Shishito	193	819	385	62	19,961
	UFO Mini Black	199	5667	1809	318	123,321
	UFO Purple	199A	1300	557	160	31,386
	Vezena Piperka	139	5	2	0	113
***C. baccatum***	Aji Ayuyo	159	940	496	189	24,877
	Aji Cambuci	56	15	8	0	370
	Aji Delight	51	0	0	0	0
	Aji Mango	171	1320	615	212	33,125
	Aji Pineapple	172	2173	1050	222	53,955
	Brazilian Starfish	176	1541	269	92	29,997
	Chinchi Amarillo	174	744	262	116	17,275
	Lemon Drop	52	2315	1131	176	57,117
	Mad Hatter	175	4	1	0	81
	Oro de Ecuador	177	625	182	123	14,137
	Sugar Rush Peach	62	580	522	100	18,672
	Sugar Drop Orange	154	1137	1023	223	36,850
	White Lightning Bolt	170	807	479	116	21,783
	White Lightning Bolt	170A	909	891	167	30,533
***C. chinense***	Aji Charapita	50	3690	466	628	72,752
	Biquinho	53	2473	419	83	47,333
	Biquinho JPM	53A	3	3	0	97
	Biquinho White	53B	1847	229	124	34,577
	Biquinho Yellow	53C	2678	669	91	54,733
	Black Panther	135	15,741	7205	365	372,825
	Carolina Reaper	160	19,636	11,969	1017	518,299
	CGN 21500	133A	10,204	2147	565	204,106
	CGN 21500	133B	8379	1672	114	162,881
	Dragon Breath	162	17,991	10,004	786	458,029
	Ghost	61A	7381	1451	100	143,125
	Ghost	61B	9027	1925	152	177,741
	Habanero	59C	11,214	1848	656	216,399
	Habanero Sweet	59B	4	0	0	64
	Hallows Eve	129	14,751	5870	327	335,039
	KS Lemon Starburst	130	10,752	2947	49	221,010
	Pink Tiger	134	7424	1234	309	142,268
	Scorpion Apocalypse	161	463	136	36	9979
	Scorpion Hulk	164	14,424	7397	305	354,155
	Scorpion Hulk	164A	10,011	4156	286	230,749
	Tobago Seasoning	156	6	2	0	129
	Viper Trinidad	163	14,143	5594	329	320,825
***C. frutescens***	Wiri Wiri	95 (137)	7718	2574	144	167,040
***C. pubescens***	Rocoto Red	49 (54)	1137	2023	223	52,950

^1^ C, capsaicin; ^2^ DHC, dihydrocapsaicin; ^3^ NDHC, nordihydrocapsaicin in μg/g dry weight. ^4^ Scolville Heat Units calculated as C*16.1 + DHC*16.1 + NDHC*9.3.

**Table 3:** Total Phenolics and Antioxidant Profiles of Chili Peppers Grown in Year 2. Different letters indicate significantly different values based on Tukey’s HSD test (*p* < 0.05).

Species	Cultivar	Internal Code	Phenolics, mg/g DW	ABTS µM TE/g DW	DPPH µM TE/g DW	FRAP µM TE/g DW
***C. annuum***	Banana Sweet	14 (136)	1.150 ± 0.079 ^kr^	11.583 ± 0.322 ^m–p^	6.097 ± 0.386 ^hp^	10.715 ± 0.439 ^jl^
	Cayenne	93 (138)	1.618 ± 0.099 ^ek^	16.167 ± 0.411 ^ik^	8.380 ± 0.404 ^bf–i^	18.421 ± 0.077 ^h^
	Jalapeno Tiger	57D	1.740 ± 0.174 ^d–i^	17.703 ± 0.393 ^h–j^	6.820 ± 0.615 ^f–o^	15.689 ± 0.302 ^i^
	Jalapeno TAM	57E	1.881 ± 0.142 ^d–g^	10.041 ± 0.109 ^nq–s^	7.091 ± 0.211 ^e-n^	9.864 ± 0.177 ^kl^
	Ethiopian Brown	201	1.602 ± 0.098 ^mr^	13.038 ± 0.362 ^l–n^	5.641 ± 0.271 ^kp^	11.887 ± 0.048 ^jk^
	Shishito	193	1.425 ± 0.015 ^gk–n^	8.021 ± 0.007 ^qw^	8.396 ± 0.047 ^bf–i^	6.631 ± 0.049 ^nq^
	UFO Mini Black	199	1.381 ± 0.029 ^hk–n^	16.472 ± 0.542 ^ik^	6.351 ± 0.397 ^fp^	12.550 ± 0.504 ^j^
	UFO Purple	199A	1.348 ± 0.083 ^nr^	11.720 ± 0.385 ^m–o^	5.848 ± 0.478 ^jp^	8.775 ± 0.148 ^ln^
	Vezena Piperka	139	1.312 ± 0.032 ^i–o^	7.490 ± 0.011 ^sw^	7.177 ± 0.083 ^e–n^	5.613 ± 0.101 ^oq–t^
***C. baccatum***	Aji Ayuyo	159	1.022 ± 0.043 ^lr^	8.066 ± 0.286 ^qw^	5.945 ± 0.093 ^ip^	4.456 ± 0.100 ^rw^
	Aji Cambuci	56	1.156 ± 0.086 ^kr^	6.460 ± 0.256 ^tw^	4.873 ± 0.985 ^np^	4.229 ± 0.035 ^rw^
	Aji Delight	51	0.879 ± 0.070 ^or^	6.077 ± 0.060 ^uw^	4.559 ± 0.378 ^op^	3.256 ± 0.028 ^vw^
	Aji Mango	171	1.089 ± 0.025 ^lr^	6.759 ± 0.640 ^tw^	5.666 ± 0.844 ^jp^	3.865 ± 0.078 ^sw^
	Aji Pineapple	172	1.142 ± 0.048 ^kr^	7.719 ± 0.449 ^rw^	5.731 ± 0.805 ^jp^	4.577 ± 0.076 ^qw^
	Brazilian Starfish	176	1.124 ± 0.121 ^lr^	6.388 ± 0.572 ^tw^	6.745 ± 0.136 ^f–o^	3.609 ± 0.057 ^tw^
	Chinchi Amarillo	174	0.862 ± 0.036 ^or^	6.350 ± 0.316 ^tw^	4.566 ± 0.911 ^op^	3.252 ± 0.058 ^vw^
	Lemon Drop	52	1.013 ± 0.097 ^lr^	7.263 ± 0.016 ^sw^	5.214 ± 0.319 ^lp^	7.194 ± 0.135 ^m–p^
	Mad Hatter	175	1.405 ± 0.028 ^gk–n^	6.305 ± 0.833 ^tw^	7.298 ± 0.385 ^d–n^	3.081 ± 0.061 ^w^
	Oro de Ecuador	177	0.664 ± 0.043 ^r^	4.995 ± 0.202 ^w^	4.861 ± 0.159 ^np^	3.210 ± 0.057 ^vw^
	Sugar Rush Peach	62	1.499 ± 0.122 ^fkl^	8.504 ± 0.108 ^p–v^	7.540 ± 0.262 ^d–l^	7.592 ± 0.055 ^m–o^
	Sugar Drop Orange	154	1.013 ± 0.089 ^lr^	7.550 ± 0.164 ^sw^	3.994 ± 0.498 ^p^	5.319 ± 0.107 ^p–v^
	White Lightning Bolt	170	0.813 ± 0.004 ^pr^	5.574 ± 0.259 ^vw^	4.040 ± 0.765 ^p^	3.196 ± 0.043 ^vw^
	White Lightning Bolt	170 A	0.799 ± 0.017 ^pr^	5.885 ± 0.067 ^vw^	4.970 ± 0.011 ^mp^	3.590 ± 0.031 ^tw^
***C. chinense***	Aji Charapita	50	1.069 ± 0.032 ^lr^	15.074 ± 0.263 ^j–l^	5.723 ± 0.075 ^jp^	11.658 ± 0.162 ^jk^
	Biquinho	53	1.496 ± 0.046 ^fkl^	17.381 ± 0.185 ^ij^	6.574 ± 0.581 ^f–o^	15.029 ± 1.109 ^i^
	Biquinho JPM	53A	1.445 ± 0.032 ^g k–m^	8.439 ± 0.5056 ^q–v^	4.952 ± 0.589 ^m p^	5.491 ± 0.092 ^o q–u^
	Biquinho White	53B	1.238 ± 0.066 ^j–q^	9.023 ± 0.032 ^oq–u^	6.565 ± 0.625 ^f–o^	8.833 ± 0.122 ^lm^
	Biquinho Yellow	53C	1.406 ± 0.049 ^g–n^	16.963 ± 0.805 ^ij^	7.898 ± 0.092 ^cf–k^	12.162 ± 0.185 ^j^
	Black Panther	135	1.291 ± 0.047 ^i–p^	32.163 ± 0.121 ^e^	5.411 ± 0.043 ^kp^	39.290 ± 0.431 ^e^
	Carolina Reaper	160	3.078 ± 0.021 ^a^	77.847 ± 0.193 ^a^	11.641 ± 0.064 ^a^	111.777 ± 0.321 ^a^
	CGN 21500	133A	1.307 ± 0.008 ^i–o^	18.996 ± 0.331 ^hi^	7.425 ± 0.047 ^d–m^	17.947 ± 0.377 ^h^
	CGN 21500	133B	1.753 ± 0.137 ^d–i^	20.567 ± 1.593 ^h^	10.252 ± 0.134 ^a–c^	18.770 ± 0.147 ^gh^
	Dragon Breath	162	2.382 ± 0.182 ^bc^	64.680 ± 0.716 ^b^	9.372 ± 0.159 ^a–e^	98.194 ± 0.361 ^b^
	Ghost	61A	2.710 ± 0.171 ^ab^	27.680 ± 0.164 ^f^	11.778 ± 0.876 ^a^	23.643 ± 0.430 ^f^
	Ghost	61B	1.722 ± 0.124 ^d–j^	26.617 ± 0.376 ^fg^	8.169 ± 0.337 ^bf–j^	25.109 ± 0.136 ^f^
	Habanero	59C	1.131 ± 0.016 ^kr^	23.975 ± 0.193 ^g^	5.049 ± 0.010 ^l–p^	20.039 ± 1.092 ^gh^
	Habanero Sweet	59B	1.070 ± 0.037 ^lr^	9.209 ± 0.472 ^oq–t^	7.156 ± 0.097 ^e–n^	6.383 ± 0.119 ^oqr^
	Hallows Eve	129	2.050 ± 0.080 ^ce^	51.604 ± 1.812 ^c^	9.737 ± 0.255 ^a–d^	54.011 ± 0.825 ^c^
	KS Lemon Starburst	130	2.141 ± 0.084 ^cd^	10.792 ± 0.322 ^mqr^	8.689 ± 0.641 ^bfg^	11.691 ± 0.242 ^jk^
	Pink Tiger	134	1.223 ± 0.056 ^k–q^	7.430 ± 0.020 ^sw^	6.211 ± 0.082 ^gp^	6.008 ± 0.056 ^oq–s^
	Scorpion Apocalypse	161	1.145 ± 0.094 ^kr^	5.626 ± 0.140 ^vw^	5.042 ± 0.219 ^lp^	3.408 ± 0.063 ^uw^
	Scorpion Hulk	164	2.681 ± 0.155 ^ab^	51.861 ± 0.878 ^c^	10.451 ± 0.272 ^ab^	55.895 ± 1.036 ^c^
	Scorpion Hulk	164A	0.980 ± 0.026 ^mr^	13.493 ± 0.418 ^km^	3.915 ± 0.119 ^p^	12.609 ± 0.260 ^j^
	Tobago Seasoning	156	0.793 ± 0.013 ^qr^	5.077 ± 0.123 ^w^	5.069 ± 0.080 ^lp^	3.485 ± 0.049 ^tw^
	Viper Trinidad	163	1.967 ± 0.051 ^cef^	43.542 ± 0.340 ^d^	8.778 ± 0.110 ^bf^	51.365 ± 0.927 ^d^
***C. frutescens***	Wiri Wiri	95 (137)	1.331 ± 0.099 ^i–o^	18.045 ± 0.259 ^h–j^	6.283 ± 0.292 ^fp^	20.761 ± 0.213 ^g^
***C. pubescens***	Rocoto Red	49 (54)	1.869 ± 0.018 ^d–h^	10.975 ± 0.218 ^mq^	8.478 ± 0.577 ^bf–h^	11.643 ± 0.151 ^jk^

## Data Availability

Data are contained within the article.
